# Impact of fixation method on femoral bone loss: a retrospective evaluation of stem loosening in first-time revision total hip arthroplasty among two hundred and fifty five patients

**DOI:** 10.1007/s00264-024-06230-4

**Published:** 2024-06-01

**Authors:** Nele Wagener, Matthias Pumberger, Sebastian Hardt

**Affiliations:** Center for Musculoskeletal Surgery, Department of Orthopaedic Surgery, Charité Universitätsmedizin Berlin, corporate member of Freie Universität Berlin and Humboldt-Universität zu Berlin, Charitéplatz 1, 10117 Berlin, Germany

**Keywords:** Femoral bone loss, Paprosky classification, Septic and aseptic loosening, Cemented versus uncemented stem fixation, First-time hip revision, Longevity of hip implants

## Abstract

**Purpose:**

Implant loosening represent the most common indication for stem revision in hip revision arthroplasty. This study compares femoral bone loss and the risk of initial revisions between cemented and uncemented loosened primary stems, investigating the impact of fixation method at primary implantation on femoral bone defects.

**Methods:**

This retrospective study reviewed 255 patients who underwent their first revision for stem loosening from 2010 to 2022, receiving either cemented or uncemented stem implants. Femoral bone loss was preoperatively measured using the Paprosky classification through radiographic evaluations. Kaplan-Meier analysis estimated the survival probability of the original stem, and the hazard ratio assessed the relative risk of revision for uncemented versus cemented stems in the first postoperative year and the following two to ten years.

**Results:**

Cemented stems showed a higher prevalence of significant bone loss (type 3b and 4 defects: 32.39% vs. 2.72%, *p* < .001) compared to uncemented stems, which more commonly had type 1 and 2 defects (82.07% vs. 47.89%, *p* < .001). In our analysis of revision cases, primary uncemented stems demonstrated a 20% lower incidence of stem loosening in the first year post-implantation compared to cemented stems (HR 0.8; 95%-CI 0.3-2.0). However, the incidence in uncemented stems increased by 20% during the subsequent years two to ten (HR 1.2; 95%-CI 0.7–1.8). Septic loosening was more common in cemented stems (28.17% vs. 10.87% in uncemented stems, *p* = .001). Kaplan-Meier analysis indicated a modestly longer revision-free period for cemented stems within the first ten years post-implantation (*p* < .022).

**Conclusion:**

During first-time revision, cemented stems show significantly larger femoral bone defects than uncemented stems. Septic stem loosening occurred 17.30% more in cemented stems.

## Introduction

Despite improved implant survival of total hip arthroplasties (THA) over the last years, the number of replacement surgeries continues to increase [1].

The most common indication for stem revision is loosening of the primary prostheses [2, 3]. This can be due to aseptic or septic conditions [4]. The most common cause of aseptic stem loosening occurs due to particle-induced reactions caused by the release of small abrasive particles, which can cause local, chronic inflammation [5, 6]. As a result of macrophage activation and induced osteoclastogenesis, peri-implant osteolysis and associated stem loosening and periprosthetic bone resorption may occur [7, 8]. During this process, a periprosthetic membrane develops between the loosened stem and the bone [9].

The incidence of periprosthetic joint infection (PJI) following primary total hip arthroplasty is approximately 1.05% in database studies and 1.74% in clinic studies [10], with a recent increase in PJI cases [11–14]. The main causes include intraoperative contamination, postoperative infection, hematogenous dissemination, chronic skin infection, and previous prosthetic infection [15, 16]. Infection can result in bone resorption, density loss, defects, and periprosthetic fractures, leading to serious complications [17, 18].

Implant fixation choice is key in hip replacement surgery, with cemented and uncemented methods available.

The use of cement might influence bone remodeling, including osteoclastogenesis, potentially leading to reduced stress and bone stimulation near the stem, which could decrease bone remodeling [19–21]. Cement can cause bone defects if unevenly distributed or degraded over time [19, 22]. However, outcomes vary among patients with cemented hip replacements, influenced by stem design, surgical technique, bone quality, and individual response [23–25].

In contrast, uncemented stems are theorized to maintain physiological stress patterns on the bone, thereby preserving remodeling activities [26, 27]. This study seeks to delineate the disparities in bone defect patterns between cemented and uncemented stems at the juncture of first-time revision.

## Materials and methods

**Fig. 1:**
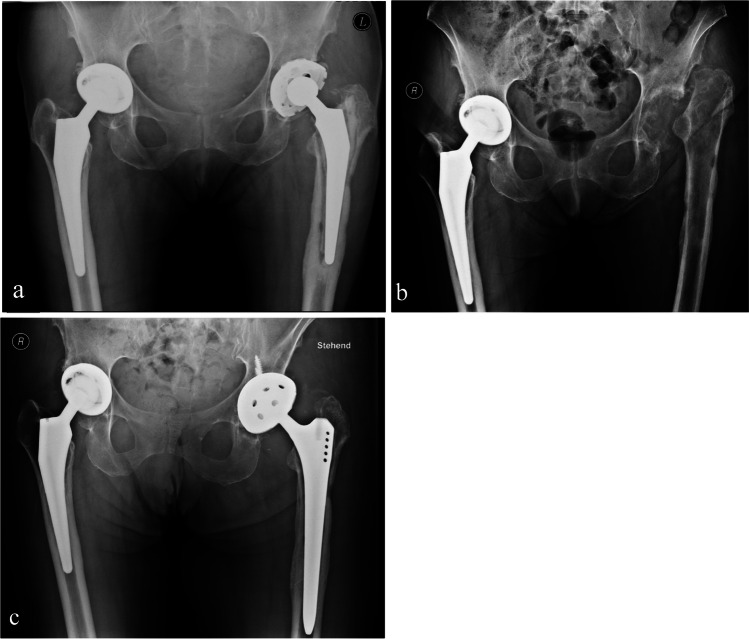
** a** depicts a case of septic loosening in a patient with a left cemented primary stem and a substantial femoral defect, classified as Paprosky 3a. This patient underwent a two-stage stem revision process. **b** shows a Girdlestone situation following the removal of a primary THA. **c** illustrates the postoperative state after the insertion of an uncemented revision stem, specified by the SLR type from Smith & Nephew, and a revision cup of the TMT type from Zimmer.

 This retrospective study, approved by our institution’s ethics committee (EA4/129/23), analyzed 255 out of 1,365 first-time revision surgeries post-primary THA from January 2010 to December 2022, including cases from both our facility and external institutions. The study focused on aseptic and septic loosening of cemented and uncemented stems. PJI was determined using EBJIS criteria [28]. Exclusions included acetabular loosening, incomplete data, periprosthetic fractures, replacement of head/inlay, metallosis, painful THA, dislocations, impingements, leg length discrepancies, implant failures, and instability. Pre-revision femoral bone loss was classified using the Paprosky et al. classification, based on surgical reports and radiographs (Fig. 1). Data collected included implant survival times, THA indications, Paprosky classifications, and patient comorbidities.

### Bone defect size assessment

**Fig. 2:**
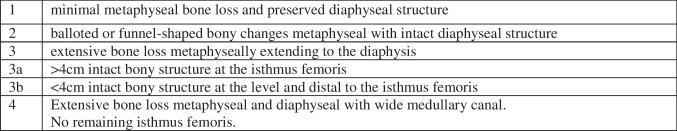
femoral bone defect size according to Paprosky et al. [29]

 Preoperative imaging studies, including pelvic overviews and axial hip radiographs taken before first-time revision surgery, were systematically reviewed. Two independent investigators, NW and SH, evaluated the size of femoral bone defects using the Paprosky classification (Fig. 2). In cases where consensus between NW and SH was not reached, a third independent surgeon was consulted.

### Statistical analysis

Categorical data were presented as frequencies and percentages, and continuous data as means and standard deviations. Associations were analyzed using chi-square or Fisher’s tests (*p* < .05). Normality of continuous variables was tested with Kolmogorov-Smirnov; normally distributed data (*p* > .05) used means, standard deviations, and parametric tests (t-Test), while non-normally distributed data used medians, quartiles, and nonparametric methods (Mann-Whitney-U test). Kaplan-Meier method was used for survival analysis, and Cox proportional hazards model adjusted for confounders. All tests were two-tailed with a 5% significance level. Analyses were conducted using SPSS version 29, IBM Inc., and R for survival analysis.

## Results

**Fig. 3:**
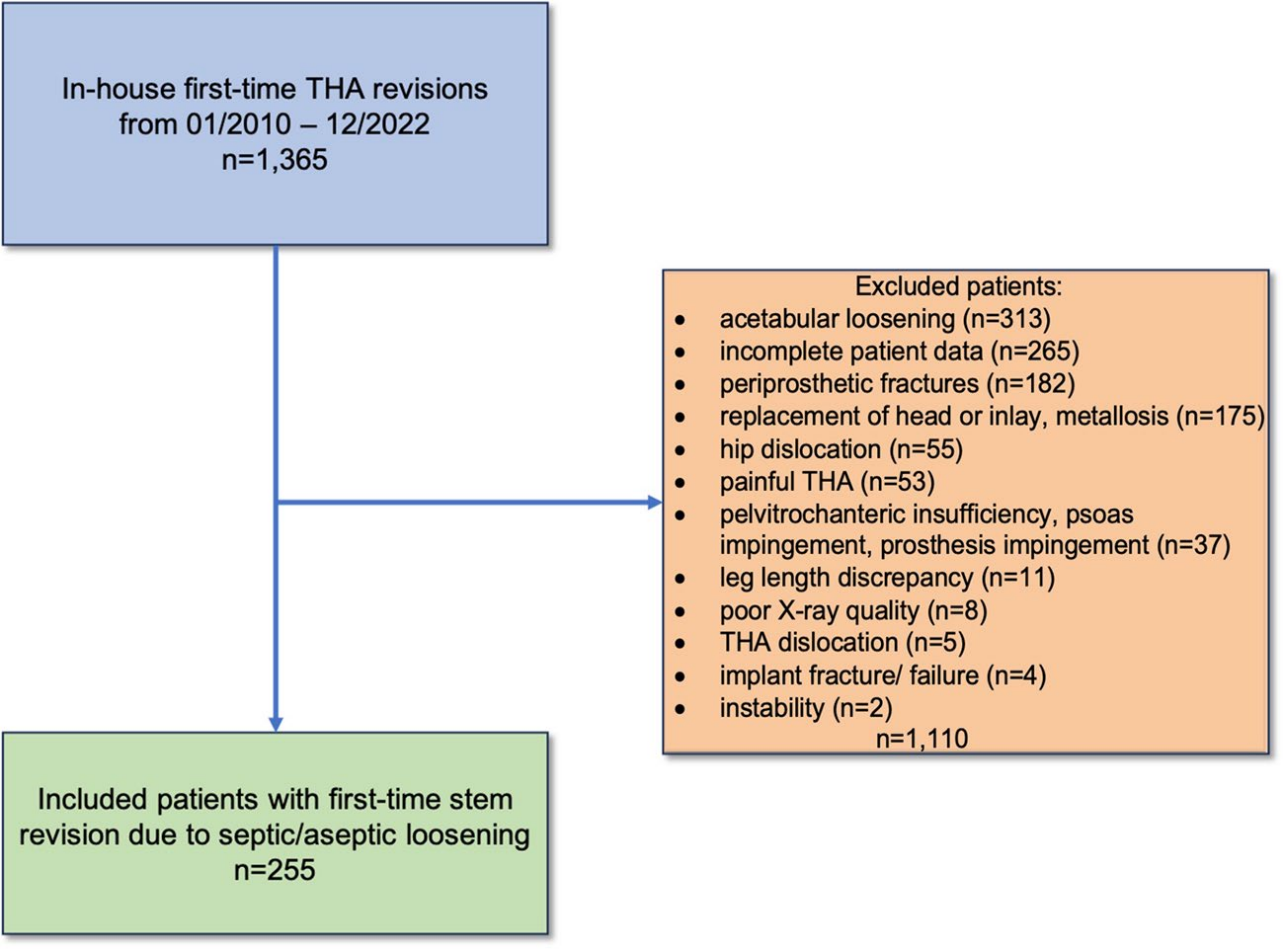
flow-chart of patient selection

Patient selection is illustrated in Fig. 3. The study encompassed 255 patients (147 females, 108 males) with an average age of 73 years at the time of their first revision surgery. The study cohort was categorized into two groups based on the nature of stem loosening: 71 patients (27.84%) with cemented stems and 184 patients (72.16%) with uncemented stems, all of whom underwent stem replacement during the initial revision period from January 2010 to December 2022.

Demographic characteristics and clinical profiles are tabulated in Table 1.

The average age at primary surgery was similar for both cemented and uncemented stem loosening groups (63 vs. 60, *p* = .455). Patients with cemented stem loosening more frequently had higher ASA scores (3–4) than those with uncemented stems (52.11% vs. 39.13%, *p* = .298). CHD was more common in the cemented group (54.93% vs. 48.91%, *p* = .389), while renal failure incidence was 6% higher in the uncemented group (13.04% vs. 7.04%, *p* = .176). The initial diagnoses of primary and secondary osteoarthritis were similarly distributed in both groups (Table 1).

## **Comparative analysis of outcomes in cemented vs. uncemented stems for primary implantation and first-time revision in hip arthroplasty**

At the time of primary implantation, osteoporosis was diagnosed in 14.08% of patients with cemented stems and 12.00% of those with uncemented stems (*p* = .646). The primary surgical approach was predominantly lateral, with 76.06% in the cemented group and 42.93% in the uncemented group (*p* < .001). Hip types indicated a majority of coxa norma with 74.65% in the cemented group versus 90.22% in the uncemented group (*p* < .001). Preoperative CCD angles averaged 125.85° for cemented and 127.38° for uncemented stems (*p* = .274). Dorr type B was most common, occurring in 91.55% of cemented stem cases and 70.11% of uncemented stem cases (*p* < .001).

During the first revision, aseptic loosening occurred more frequently in patients with uncemented stems compared to those with cemented stems (60.33% vs. 32.39%, *p* < .001). Conversely, septic loosening occurred 17.3% more often in the cemented stem group than in the uncemented cohort (28.17% vs. 10.87%, *p* = .001), while patients with cemented stems showed concurrent cup loosening more frequently than patients with uncemented stems (39.44% vs. 28.8%, *p* = .105) (Table 1). Cemented stems had a longer implant survival (mo.) than uncemented stems and were consequently revised for the first time later (143 vs. 90, *p* = .009). Furthermore, the mean operative time for the first revision procedure was extended by 37 min for those with cemented stems (165 vs. 128, *p* < .001).

**Table 1:** Characteristics of the study population and first-time revision

		Cemented *N* = 71	Uncemented *N* = 184	*p*-value
Primary Implantation				
Age at primary implantation		63 (49; 71)	60 (42; 68)	0.455
Sex	Female/Male	53/18 (74.65%)	94/90 (51.09%)	0.001
BMI		26 (23; 31)	28 (25; 31)	0.184
ASA	1	7 (9.86%)	25 (13.59%)	0.298
	2	27 (38.03%)	87 (47.28%)	
	3	36 (50.70%)	70 (38.04%)	
	4	1 (1.41%)	2 (1.09%)	
Osteoporosis	Yes/No	10/61 (14.08%)	22/162 (12.00%)	0.646
Rheumatoid arthritis	Yes/No	4/67 (5.63%)	8/176 (4.35%)	0.664
CHD	Yes/No	39/32 (54.93%)	90/94 (48.91%)	0.389
COPD	Yes/No	6/65 (8.45%)	13/171 (7.07%)	0.706
Gastric ulcer	Yes/No	2/69 (2.82%)	2/182 (1.09%)	0.319
Liver disease	Yes/No	4/67 (5.63%)	12/172 (6.52%)	0.793
Apoplexy	Yes/No	3/68 (4.23%)	6/178 (3.26%)	0.708
Dementia	Yes/No	1/70 (1.41%)	2/182 (1.09%)	0.831
PAD	Yes/No	2/69 (2.82%)	5/179 (2.72%)	0.965
Diabetes mellitus	Yes/No	5/66 (7.04%)	22/162 (11.96%)	0.253
Oncological disease	Yes/No	7/64 (9.86%)	27/157 (14.67%)	0.311
Metastatic disease	Yes/No	1/70 (1.41%)	1/183 (0.54%)	0.483
Renal failure	Yes/No	5/66 (7.04%)	24/160 (13.04%)	0.176
Hypothyroidism	Yes/No	7/64 (9.86%)	22/162 (11.96%)	0.636
AIDS	Yes/No	0/71 (0%)	1/183 (0.54%)	0.534
Smoking	Yes/No	11/60 (15.49%)	28/156 (15.22%)	0.956
Alcohol	Yes/No	11/60 (15.49%)	22/162 (11.96%)	0.451
Diagnosis at primary THA	Primary osteoarthritis	60 (87.14%)	157 (85.33%)	0.175
	Secondary osteoarthritis	9 (12.68%)	27 (14.67%)	
Surgical approach	Anterolateral Lateral Posterolateral	0 (0%) 54 (76.06%) 17 (23.94%)	89 (48.37%) 79 (42.93%) 16 (8.70%)	< 0.001
Duration of surgery (min.)		62.41 ± 5.20	49.60 ± 7.13	< 0.001
Hip type	Coxa vara (CCD < 120°) Coxa norma (CCD 120–140°) Coxa valga (CCD > 140°)	125.85 ± 6.55	127.38 ± 11.05	0.274
Dorr type A B C		0 (0%) 65 (91.55%) 6 (8.45%)	54 (29.35%) 129 (70.11%) 1 (0.54%)	< 0.001
First-Time Revision				
Age at 1st time revision		76 (70; 81)	70 (63; 76)	< 0.001
Implant survival (mo.)		143 (46; 282)	90 (36; 150)	0.009
Aseptic stem loosening		23 (32.39%)	111 (60.33%)	< 0.001
Septic stem loosening		20 (28.17%)	20 (10.87%)	0.001
THA loosening		28 (39.44%)	53 (28.8%)	0.105
Type of revision stem replacement		19 (26.76%)	91 (49.46%)	0.001
THA replacement		52 (73.24%)	93 (50.54%)	
Surgery duration of 1st time revision (min.)		165 (130; 213)	128 (101; 172)	< 0.001

Table 1 Demographics for metric variable age (normally distribution cannot be assumed) is presented as median (lower; upper quartile), for categorical variables as total numbers and frequencies. *PAD* peripheral arterial disease, *BMI* body-mass-index (kg/m2), *CHD* coronary heart disease, *COPD* chronic obstructive pulmonary disease, *ASA* american society of anesthesiologists. Comparison of parameters of first-time revision between cemented and uncemented stems. Body-mass-index (kg/m2) (BMI); mo. (months); THA (Total hip arthroplasty). P-values resulting from Chi-Square test or Fisher exact test for categorical variables and Mann-Whitney test or t-Test for age, duration of primary surgery or preoperative CCD angle. NA: not applicable due to zero cell frequencies

### Predominance of cemented and distal-anchored prostheses in initially cemented stems

In stem revisions, implant choice is influenced by bone defect size. Patients with original cemented stems often received another cemented implant or a distal-anchored revision stem. Data showed distinct implant preferences: 28.17% with initially cemented stems received a cementless Revitan stem versus 14.29% with uncemented stems (*p* = .009). Conversely, 18.31% with primary cemented stems got an uncemented SL-Plus-MIA stem, compared to 38.46% for primary uncemented stems (*p* = .003). Cemented VerSys stems were utilized in 16.90% of revisions involving primary cemented stems, as opposed to 1.10% of revisions for primary uncemented stems (*p* < .001). Similarly, 16.90% of initially cemented stems had a cementless SLR revision, rising to 29.12% for primary uncemented stems (*p* = .051). The cemented SPII Lubinus stem was chosen in 11.27% of cases with initially cemented stems, against 2.20% in uncemented (*p* = .002). This indicates a trend towards repeating cemented implants in patients with original cemented stems, while those with primary uncemented stems preferred uncemented revisions (Table 2).

**Table 2:** Characteristics of revision arthroplasty and size of femoral bone defects between cemented and uncemented stems at first-time revision

		Cemented stem in primary surgery *N* (%)	Uncemented stem in primary surgery *N* (%)		
				*p-value*	*p-value (overall)*
Revision stem brand	Revitan (uncemeted)	20 (28.17%)	26 (14.29%)	0.009	< 0.001
	SL-Plus-MIA (uncemented)	13 (18.31%)	70 (38.46%)	0.003	
	VerSys (cemented)	12 (16.90%)	2 (1.10%)	< 0.001	
	SLR (uncemented)	12 (16.90%)	53 (29.12%)	0.051	
	SPII Lubinus (cemented)	8 (11.27%)	4 (2.20%)	0.002	
	Alloclassic Zweymüller (uncemented)	3 (4.23%)	19 (10.44%)	0.120	
	Bicontact (cemented/uncemented)	2 (2.82%)	2 (1.10%)	0.319	
	Megasystem-C (uncemented)	1 (1.41%)	1 (0.55%)	0.483	
	Wagner SL (uncemented)	0	4 (2.20%)	NA	
	TRJ (uncemented)	0	1 (0.55%)	NA	
Femoral bone defects according to Paprosky et al.	Paprosky I and II	34 (47.89%)	151 (82.07%)	< 0.001	< 0.001
	Paprosky IIIA	14 (19.72%)	28 (15.22%)	0.385	
	Paprosky IIIB and IV	23 (32.39%)	5 (2.72%)	< 0.001	
	I	19 (26.76%)	69 (37.50%)	0.106	< 0.001
	II	15 (21.13%)	82 (44.57%)	0.001	
	IIIA	14 (19.72%)	28 (15.22%)	0.385	
	IIIB	21 (29.58%)	5 (2.72%)	< 0.001	
	IV	2 (2.82%)	0	NA	

*P*-values resulting from Chi-Square test. NA: not applicable due to zero cell frequencies

**Table 3:** Size of femoral bone defects between cemented septic and cemented aseptic stems at first-time revision

Cemented stem loosening			septic	aseptic		*p*-value		
			*N* (%)	*N* (%)		(overall)		
Femoral bone defects according to Paprosky et al.	I		15 (75.00%)	4 (7.84%)		< 0.001		
	II		2 (10.00%)	13 (25.49%)				
	IIIA		2 (10.00%)	12 (23.53%)				
	IIIB		1 (5.00%)	20 (39.22%)				
	IV		0 (0.00%)	2 (3.92%)				

*P*-values resulting from Chi-Square test

**Table 4:** Size of femoral bone defects between uncemented septic and uncemented aseptic stems at first-time revision

Uncemented stem loosening		septic	aseptic	*p*-value
		*N* (%)	*N* (%)	(overall)
Femoral bone defects according to Paprosky et al.	I	3 (15.00%)	66 (40.24%)	0.002
	II	9 (45.00%)	73 (44.51%)	
	IIIA	5 (25.00%)	23 (14.02%)	
	IIIB	3 (15.00%)	2 (1.22%)	
	IV	0 (0.00%)	0 (0.00%)	

*P*-values resulting from Chi-Square test

### Increased femoral bone resorption associated with cemented primary stem fixation

An evaluation of femoral bone integrity revealed that cemented stems exhibit a significantly higher prevalence of severe bone defects, with Paprosky et al.‘s type 4 and 3b defects observed in 32.39% of cases, in stark contrast to a mere 2.72% in uncemented stems before first-time revision (*p* < .001) (Table 2). The incidence of type 3a femoral bone defects was noted in 19.72% of cemented stems, compared to 15.22% associated with loosening of uncemented stems (*p* = .385). Conversely, the uncemented group demonstrated a predominance of milder type 1 and 2 defects, accounting for 82.07%, whereas such defects in the cemented cohort were noted in only 47.89% of cases (*p* < .001). This data highlights a distinct pattern and severity of bone loss that could be attributed to the method of stem fixation, both in aseptic and septic loosening conditions. Notably, cementation shows a significant correlation with advanced bone defect classes prior to initial revision surgery.

**Fig. 4:**
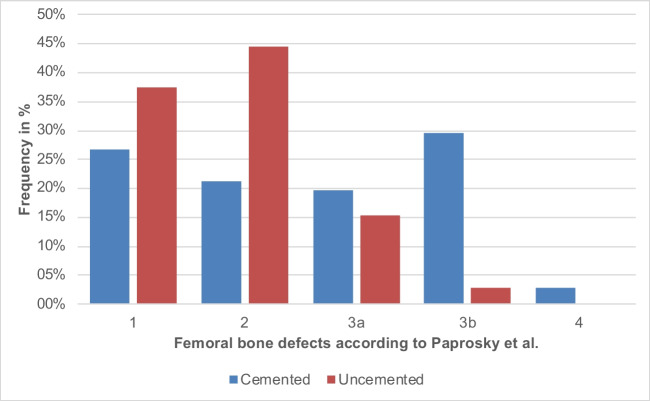
Bone defect size between cemented and uncemented stems

Our subanalysis showed that, under aseptic conditions, cemented stems exhibited severe bone defects (Paprosky type IIIB and IV) significantly more often—occurring in 43.14% of cases—compared to only 1.22% in uncemented stems (see Tables 3 and 4). The analysis of Paprosky type IIIA defects also revealed differences, type IIIA defects occurred in 23.53% of aseptic cemented stems, while they were found in 14.02% of cases in uncemented stems. Milder defects (Paprosky type I and II) were much more common in the group of uncemented stems under aseptic conditions, at 84.75% compared to 33.33% in cemented stems (Fig. 4).

### Cemented stem fixations demonstrate superior long-term implant survival

**Fig. 5:**
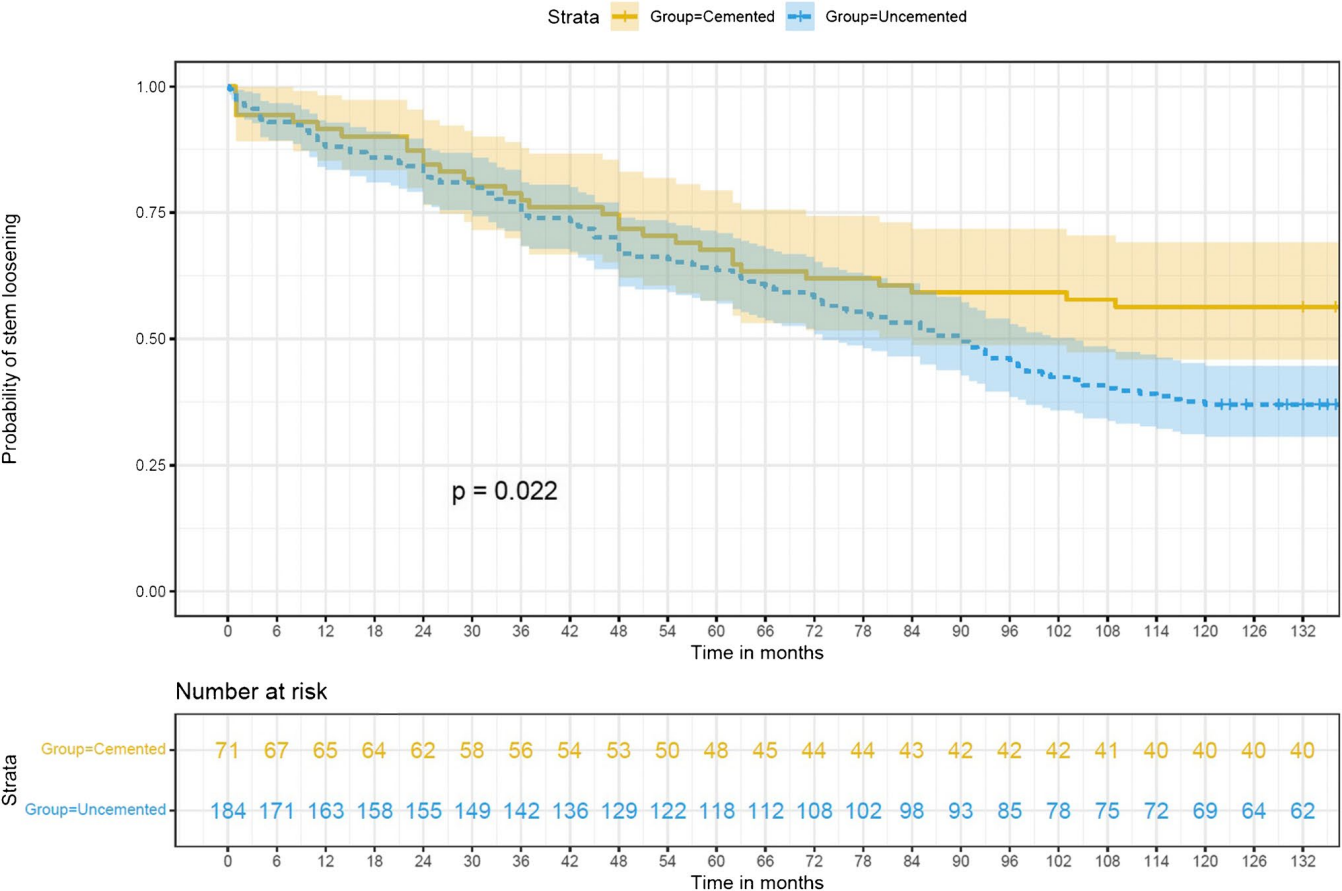
illustrates the comparative survival rates of stem implants based on fixation type, up to the point of first-time revision, with a focus on aseptic/septic stem loosening as the primary endpoint

In our analysis, the 10-year unadjusted implant survival probability, with the endpoint being the first-time revision due to septic or aseptic stem loosening, was markedly superior in cemented primary stems as opposed to uncemented ones. Specifically, cemented stems exhibited a survival probability of 0.6 (95%-Confidence Interval [CI]: 0.45–0.68), compared to 0.4 (95%-CI: 0.30–0.44) for uncemented stems, as shown in Fig. 5 (*p* < .022).

Furthermore, during the initial nine years following primary implantation, the adjusted risk of first-time revision for septic or aseptic loosening was observed to be greater in uncemented stems, though with overlapping confidence intervals. After nine years following implantation, the adjusted survival probability of cemented stems tends to be higher compared to that of uncemented stems.

Kaplan Meier (KM) curves (95% CI) and Log-rank test for survival rate of the stem over grouped factors. The KM survival curves for each grouped factor were identified by colour and pattern differences.

Our data indicates a different risk profile for uncemented versus cemented stem designs in aseptic/septic stem revisions after primary implantation. Uncemented stems show a lower risk of first-time revision within the first year post-implantation, with a hazard ratio (HR) of 0.8 and a 95%-confidence interval (95%-CI) of 0.3 to 2.0, as seen in Table 5. However, from the second to the tenth year post-implantation, uncemented stems have a higher risk of first-time revision. This period sees a 1.2 times increased risk compared to cemented stems, with an HR of 1.2 and a 95%-CI of 0.7–1.8.

**Table 5:** Hazard ratio (HR) for first-time revision due to stem loosening of cemented and uncemented primary stems

		HR (95%-CI)	*p*-value
Uncemented vs. Cemented	1 year revision	0.8 (0.3–2.0)	0.62
	2–10 year revision	1.2 (0.7–1.8)	0.528

The Cox regression was adjusted for age, sex, and femoral bone defect size (aggregating defects into 3 main groups along Paprosky classes 1 + 2, 3a, 3b + 4). Cox-regression hazards ratio (HR) in multivariate analysis for predicting time to first-time revision due to aseptic stem loosening.

## Discussion

The optimal fixation method for primary total hip arthroplasty—cemented or uncemented—continues to be a subject of clinical debate [30]. In our patient cohort, cemented stems were associated with a 32.39% incidence of significant bone defects (types 3b and 4) at the time of first revision. In contrast, such defects were observed in only 2.72% of cases with uncemented stems. When considering minor bone defects (types 1 and 2), uncemented stems were predominant, accounting for 82.07% in comparison to 47.89% for cemented stems. Moreover, a 17.30% greater occurrence of septic loosening was noted with cemented stems relative to their uncemented equivalents (28.17% vs. 10.87%, *p* < .001).

This observation aligns with findings from Tyson et al., who reported similar patterns of bone defect size in a registry study involving aseptic loosening after initial revision with uncemented/cememted fixation [22]. Gromov et al. also corroborated the trend of more severe bone defects being associated with cemented femoral components at re-revision [31]. Contrasting with the findings of Tyson et al. and Gromov et al., our investigation assessed femoral bone defects prior to the first revision and discerned that larger defects were also present with cemented stems. The literature suggests that for patients presenting with extensive bone defects and porous or osteoporotic bone, revision arthroplasties tend to be cemented or diaphyseally fixed to provide stable fixation across the defect site [32, 33]. Conversely, for those with robust bone quality and minor defects, an uncemented press-fit approach is favored to facilitate the biological integration of the implant through bone ongrowth [34]. Our study further substantiates this practice, indicating that patients initially receiving cemented stems typically underwent cemented or distally fixed revisions, implying the presence of larger bone defects [35, 36]. This finding raises particular concern for younger patients who are more likely to undergo future revisions; hence, the preservation of bone stock and the utilization of bone-sparing techniques are of the utmost importance. However, the selection of implants for initial revision procedures depends not only on the type of primary fixation, but also on a variety of other factors. These include the extent of the bone defect, the quality of the patient’s bone, the patient’s age, and their level of physical activity or lifestyle demands [31]. Additionally, the patient’s overall health status, the presence of any comorbidities, and the stability of the surrounding soft tissue structures play crucial roles in the decision-making process [37–39]. In the context of our study, the evaluation of osteoporosis prevalence at the time of primary implantation revealed no significant differences between the primary cemented and uncemented stem fixation groups within our cohort. This finding is crucial as it highlights the nuanced considerations required in choosing the appropriate stem fixation method. Moreover, the dominance of coxa norma in both cemented and uncemented groups underscores the commonality of hip geometry across different fixation types. Additionally, the prevalence of Dorr Type B among patients, irrespective of the stem fixation method, points to a predominant bone quality pattern in our cohort. These observations provide valuable insights into the factors influencing the choice of stem fixation and its potential impact on surgical outcomes, suggesting that both cemented and uncemented stems can be suitable for a wide range of bone qualities and hip geometries, contingent upon careful preoperative evaluation. Reflecting on the bone quality and shape of the proximal femur, as well as the angular relationships between the neck and shaft of the femur, which influence the biomechanics and loading of the hip joints, it was observed that these characteristics were approximately equally distributed at the time of primary implantation across both patient groups (cemented and uncemented stem fixation). This distribution suggests that the larger bone defects observed in our cohort may be more attributable to the use of cemented stem fixation. Furthermore, the surgeon must consider the likelihood of future revisions, the ease of implantation, and the expected longevity of the implant based on the patient’s life expectancy. These considerations ensure that the chosen implant best suits the individual needs and circumstances of each patient.

Cemented hip stems utilize Polymethyl methacrylate (PMMA) bone cement to secure the prosthesis stem within the bone. Over time, potential loosening of this cement can lead to implant instability and the enlargement of bone defects [20, 40]. Bone cement may also provoke a biological response where the body attempts to resorb or bypass the material, exacerbating bone loss [40, 41]. Furthermore, cement degradation and the consequent peri-implant osteolysis due to the immunogenic reaction to cement particles can precipitate aseptic inflammation, further compromising peri-implant bone integrity [42–44].

In their analytical study, Gromov et al. reported a predominance of “aseptic stem loosening” as a revision cause, attributing 74% to cemented stems as opposed to 25% [31]. Diverging from Gromov’s findings, our data indicated a 28% elevated rate of aseptic loosening in uncemented stems and a 17.3% increased incidence of septic loosening in cemented stems. Uncemented stems rely on bone growth into the implant for stability, and this process can be affected by the mechanical stresses from physical activities [45]. Over time, these stresses might lead to micro-movements between the bone and the implant, potentially causing aseptic loosening [46, 47]. Conversely, the loosening rate in cemented stems could be linked to the inherent properties of cement, which may deteriorate or become unstable over time, thereby escalating infection risks. Within our patient population, the incidence of septic loosening in cemented stems was notable, comprising 17.3% of cases, suggesting an association between cementation and heightened infection susceptibility. The study “Two-stage revision for periprosthetic joint infection in cemented total hip arthroplasty: an increased risk for failure?” suggests that patients undergoing removal of cemented THA had higher rates of reinfection (22% compared to 7%, *p* = .021) and all-cause revision (31% compared to 14%, *p* = .039) than those with cementless THA [48]. This indicates a potential association between cementation and increased susceptibility to infection​.

Cement’s propensity to ensnare bacteria and provide a conducive environment for bacterial colonization poses a significant risk, particularly if pathogenic organisms persist in the cement post-revision surgery, potentially leading to subsequent infections [49–51]. Therefore, in septic revision scenarios, cement avoidance is commonly advocated to enhance infection management [52, 53]. Moreover, our study unveiled an estimated Hazard Ratio for first-time revision that was 0.2 times lower for uncemented stems compared to cemented ones within the initial year post-implantation. However, in the span between the second and tenth years, the risk for first-time revision was observed to be 1.2 times higher for uncemented stems. This aligns with Tyson et al.‘s findings, which identified lower ten year implant survival rates associated with uncemented stems when factoring in re-revision for any cause [22].

While the retrospective nature of our study limits the availability of detailed primary surgery data, it uniquely explores the relationship between fixation techniques and femoral bone defect size, revision probability, and implant longevity in both aseptic and septic stem loosening cases. This is the first study to compare fixation effects on femoral bone defect size, revision risk, and implant longevity in aseptically and septically loosened stems following primary THA.

Our findings underscore the complexity of choosing between cemented and uncemented stems, highlighting that this decision should not be based solely on the fixation method but must consider a myriad of factors including the patient’s age, bone quality, activity level, and comorbid conditions. Our investigation indicates that uncemented stems are linked with a higher occurrence of minor bone defects and aseptic loosening. In contrast, it has been observed that cemented stems are associated with larger bone defects and an increased risk of septic loosening. This suggests that each fixation method has its unique advantages and limitations, which must be carefully weighed against the patient’s specific clinical context. Furthermore, the study highlights the need for continued research and development in implant technology, particularly in addressing the challenges associated with cement degradation and the risk of infection in cemented stems. In the end, the decision on the type of fixation should be tailored to each patient’s individual needs, taking into account their overall health, bone condition, and lifestyle.

## Conclusion

This study concludes that cemented primary stems are associated with more extensive femoral bone defects at first-time revision and a higher incidence of septic loosening compared to uncemented stems. This differential in outcomes highlights the importance of individualized patient evaluation in choosing the appropriate fixation method for primary THA.
